# Synthesis and carbonic anhydrase activating properties of a series of 2-amino-imidazolines structurally related to clonidine^1^

**DOI:** 10.1080/14756366.2020.1749602

**Published:** 2020-04-27

**Authors:** Niccolò Chiaramonte, Soumia Maach, Caterina Biliotti, Andrea Angeli, Gianluca Bartolucci, Laura Braconi, Silvia Dei, Elisabetta Teodori, Claudiu T. Supuran, Maria Novella Romanelli

**Affiliations:** Section of Pharmaceutical and Nutraceutical Sciences, Department of Neuroscience, Psychology, Drug Research and Child’s Health, University of Florence, Sesto Fiorentino, Italy

**Keywords:** Carbonic anhydrase, activator, clonidine, histamine, imidazoline

## Abstract

The Carbonic Anhydrase (CA, EC 4.2.1.1) activating properties of histamine have been known for a long time. This compound has been extensively modified but only in few instances the imidazole ring has been replaced with other heterocycles. It was envisaged that the imidazoline ring could be a bioisoster of the imidazole moiety. Indeed, we report that clonidine, a 2-aminoimidazoline derivative, was found able to activate several human CA isoforms (hCA I, IV, VA, VII, IX, XII and XIII), with potency in the micromolar range, while it was inactive on hCA II. A series of 2-aminoimidazoline, structurally related to clonidine, was then synthesised and tested on selected hCA isoforms. The compounds were inactive on hCA II while displayed activating properties on hCA I, VA, VII and XIII, with K_A_ values in the micromolar range. Two compounds (**10** and **11**) showed some preference for the hCA VA or VII isoforms.

## 1. Introduction

Carbonic anhydrases (CAs, EC 4.2.1.1) are metallo-enzymes widespread in all life kingdoms. These enzymes catalyse a plethora of reactions, among which the reversible hydration of CO_2_ is the most important one^1^. The active site contains the cofactor, a metal ion (usually Zn^2+^) which coordinates a water molecule responsible, once activated as hydroxide ion, of the nucleophilic attack onto carbon dioxide. Eight genetically different families have been found (α-ι); 15 isoforms belonging to the α class have been characterised in humans^1^^,^^2^.

CAs have been drug targets since more than 70 years; inhibitors of these enzyme are used for the treatment of oedema, glaucoma and epilepsy but several new therapeutic applications are under study^3^. In recent years, the attention has been focussed also on activators of these enzymes, despite the fact that CA are among the most efficient enzymes known. In fact, genetic deficiencies of several CA isoforms were reported in the last decades (reviewed in Refs. [3,4]), and in principle a loss of function of these enzymes could be treated with CA selective activators (CAAs). In addition, there is evidence that CA activation improves cognitive performance^5–10^. However, the influence of CA on these processes is complex since also inhibitors have been found to improve memory deficits in animal models (reviewed in Ref. [11]); these findings point out the need for isoform selective inhibitors or activators to elucidate the role of CA isoforms in cognitive processes. Other possible applications of CAAs could be in the formation of artificial tissues^12^ and in CO_2_ capture and sequestration processes^13^.

**Chart 1.**
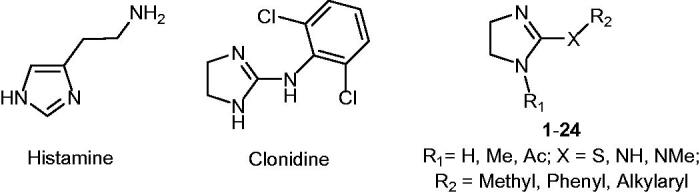
Chemical structure of CA activators.

Histamine (HST, Chart 1) was among the first reported activators, whose interaction mode was elucidated by means of X-ray crystallography^14^. The adduct with hCA II revealed a complex network of H-bonds involving the Zn-bound water molecule, His64 and the imidazole ring of the activator, which is located far away from the metal ion, in a region approaching the edge of the active site cavity. X-ray crystallographic studies have later shown that also other activators bind in this area^4^.

As common structural feature, CAAs possess flexible tails decorated with protonable moieties, with p*K*_a_ values spanning between 6 and 8. The molecule of histamine has been extensively modified, placing substituents on the imidazole C atoms and on the NH_2_ group, showing that the latter is not essential, since it can be largely modified to keep or improve potency (reviewed in Ref. [4]). Only in few instances the imidazole ring has been replaced by another heterocycle, such as a thiadiazole ring^15^.

In search for bioisosters of the imidazole moiety, our attention was attracted by the imidazoline ring. This feature is present in a well-known drug, Clonidine (CLO, Chart 1), which is clinically used as an antihypertensive agent being an agonist at the central α2-adrenergic receptor, but it is able to interact with other targets, such as the imidazoline binding sites and the hyperpolarization-activated cyclic nucleotide gated channels^16^^,^^17^. Therefore, we decided to measure the potential CA activating properties of this compound, finding that CLO behaves as CAA on several CA isoforms (Table 1). Encouraged by this positive outcome, we synthesised a series of 2-substituted imidazolines (compounds **1**–**24**, Chart 1) and tested their activity on five different hCA isoforms. The ubiquitous cytosolic enzymes CA I and II, the mitochondrial CA VA, which is associated with the glucose homeostasis,^18^ the cytosolic CA VII which is particularly abundant in the CNS and has been recently demonstrated to have a protective role against oxidative damage,^19^ and the cytosolic CA XIII, which is particularly expressed in the reproductive organs^20^^,^^21^ were selected.

**Table 1.** Activation constants of Clonidine (CLO) and histamine (HST) on selected human CA isoforms, measured by means of a stopped-flow, CO_2_ hydrase assay.^a^

Compound	hCA K_A_ (µM)							
	I	II	IV	VA	VII	IX	XII	XIII
CLO	73.6	>200	132	42.6	8.4	54.1	126	7.8
HST^b^	2.1	125	25.3	0.01	37.5	35.1	27.9	4.6

^a^Errors (data not shown) are in the range of ±5–10% of the reported values from three different assays.

^b^From Ref. [4].

## 2. Materials and methods

### 2.1. Chemistry

All melting points were taken on a Büchi apparatus and are uncorrected. NMR spectra were recorded on a Brucker Avance 400 spectrometer (400 MHz for ^1^H NMR, 100 MHz for ^13 ^C). Chromatographic separations were performed on a silica gel column by gravity chromatography (Kieselgel 40, 0.063–0.200 mm; Merck) or flash chromatography (Kieselgel 40, 0.040–0.063 mm; Merck). Yields are given after purification, unless differently stated. When reactions were performed under anhydrous conditions, the mixtures were maintained under nitrogen. High-resolution mass spectrometry (HR-MS) analyses were performed with a Thermo Finnigan LTQ Orbitrap mass spectrometer equipped with an electrospray ionisation source (ESI). Analyses were carried out in positive ion mode monitoring protonated molecules, [M + H]^+^ species, and a proper dwell time acquisition was used to achieve 60,000 units of resolution at Full Width at Half Maximum (FWHM). Elemental composition of compounds were calculated on the basis of their measured accurate masses, accepting only results with an attribution error less than 5 ppm and a not integer RDB (double bond/ring equivalents) value, in order to consider only the protonated species^22^. Compounds were named following IUPAC rules by means of ChemDraw 14.0.

#### 2.1.1. General procedure for the preparation of 2-amino-imidazoline 2–20

A mixture of the appropriate intermediate (**1a**^23^ or **1b**^24^, 0.05 g) and the amine (1 eq) was suspended in THF (5 ml) and the mixture was heated at 70 °C until reaction completion (TLC monitoring, eluent CH_2_Cl_2_/CH_3_OH/NH_4_OH 90:10:1); alternatively, an excess of amine (5 eq) was used as solvent. Volatiles were evaporated under vacuum and the residue was triturated with Et_2_O to remove the unreacted amine, affording the desired imidazoline derivative. In some instances, additional purification by means of flash chromatography was necessary. The conditions for each compound are reported in Table 2. With this method, the following compounds were prepared.

**Table 2.** Synthetic details for the preparation of compounds **2–20** (see general formula in Scheme 1) starting from **1a** (R_1_ = H) or **1 b** (R_1_ = Me) and amines R_2_R_3_NH.

N	Starting material	R_2_	R_3_	Solvent	Reaction time (h)^a^	Yields (%)^b^
**2**	**1a**	H	–CH_2_Ph	THF	7	94
**3**	**1a**	H	–(CH_2_)_2_Ph	THF	16	90
(*S*)-**4**	**1a**	H	(*S*)–CH(Me)Ph	Amine^c^	2	36^d^
(*R*)-**4**	**1a**	H	(*R*)–CH(Me)Ph	Amine^c^	2	74^d^
**5**	**1a**	H	–(CH_2_)_3_Ph	THF	3	88
**6**	**1a**	Me	–CH_2_Ph	THF	22	32^d^
**7**	**1a**	Me	–(CH_2_)_2_Ph	THF	2	88
**8**	**1b**	H	–(CH_2_)_2_Ph	Amine^c^	2	86
**9**	**1b**	Me	–(CH_2_)_2_Ph	Amine^c^	2	97
**10**	**1b**	Me	–CH_2_Ph	Amine^c^	3	70^d^
**11**	**1b**	H	–CH_2_Ph	Amine^c^	2	81
**12**	**1b**	H	–(CH_2_)_3_Ph	Amine^c^	2	45^d^
**13**	**1b**	H	–CH_2_C_6_H_4_Cl(4)	THF	16	97
**14**	**1b**	H	–CH_2_C_6_H_4_OCH_3_(4)	THF	4	89^d^
**15**	**1b**	H	–CH_2_C_6_H_4_F(4)	Amine^c^	6	89
**16**	**1b**	H	–CH_2_C_6_H_4_Cl(3)	Amine^c^	6	88^d^
**17**	**1b**	H	–CH_2_C_6_H_4_OCH_3_(3)	Amine^c^	24	92
**18**	**1b**	H	–CH_2_C_6_H_4_ F(3)	Amine^c^	16	87
**19**	**1a**	H	–(CH_2_)_2_NHCH_2_Ph	Amine^c^	3	56^d^
**20**	**1b**	H	–(CH_2_)_2_NHCH_2_Ph	Amine^c^	4	57^d^

^a^ Heating at 70 °C; all amines were commercially available.

^b^ Unless otherwise stated, yields are given after trituration with diethyl ether.

^c^ The amine was used in excess (5 eq).

^d^ After chromatographic separation.

*N*-benzyl-4,5-dihydro-1*H*-imidazol-2-amine hydroiodide **2**^23^. White solid; m.p. 143–146 °C ESI-LC-MS (*m/z*) 176.0 [M + H]^+^ [^1^H]-NMR (D_2_O) δ: 3.56 (s, 4H, CH_2_CH_2_); 4.31 (s, 2H, CH_2_); 7.24–7.35 (m, 5H, Ar) ppm. [^13^C]-NMR (D_2_O) δ: 42.70 (CH_2_Ph); 45.65 (2CH_2_); 126.93 (CH_Ar_); 128.00 (CH_Ar_); 128.95 (CH_Ar_); 136.25 (C_Ar_); 159.91 (C=N) ppm.

*N*-Phenethyl-4,5-dihydro-1*H*-imidazol-2-amine hydroiodide **3**^25^. White solid; m.p 82–83 °C; ESI-LCMS (*m/z*) 189.7 [M + H]^+^. [^1^H]-NMR (D_2_O) δ: 2.75 (t, *J* = 6.6 Hz, 2H, CH_2_); 3.33 (t, *J* = 6.6 Hz, 2H, CH_2_); 3.43 (s, 4H, CH_2_CH_2_); 7.14–7.31 (m, 5H, Ar) ppm. [^13^C]-NMR (D_2_O) δ: 34.76 (CH_2_); 42.63 (2CH_2_); 43.78 (CH_2_); 126.94 (CH_Ar_); 128.84 (CH_Ar_); 129.13 (CH_Ar_); 138.50 (C_Ar_); 159.83 (C=N) ppm.

(*S*) *N*-(1-Phenylethyl)-4,5-dihydro-1*H*-imidazol-2-amine hydroiodide (*S*)-**4**^26^. Purification by flash chromatography (CH_2_Cl_2_/CH_3_OH/NH_3_ 87:13:1.3 as eluent); m.p. 103–107 °C; ESI-LCMS (*m/z*) 190.2 [M + H]^+^. [^1^H]-NMR (D_2_O) δ: 1.41 (d, *J* = 6.8 Hz, 3H, CH_3_); 3.49–3.53 (m, 4H, 2CH_2_); 4.55 (q, *J* = 6.8 Hz, 1H, CH); 7.23–7.35 (m, 5H, Ar) ppm. [^13^C]-NMR (D_2_O) δ**:** 22.52 (CH_3_); 42.62 (2CH_2_); 52.79 (CH); 125.59 (CH_Ar_); 128.03 (CH_Ar_); 129.08 (CH_Ar_); 142.03 (C_Ar_); 158.98 (C=N) ppm.

(*R*) *N*-(1-Phenylethyl)-4,5-dihydro-1*H*-imidazol-2-amine hydroiodide (*R*)-**4**^26^. Purification by flash chromatography (CH_2_Cl_2_/CH_3_OH/NH_3_ 87:13:1.3 as eluent); m.p. 105–109 °C; ESI-LCMS (*m/z*) 190.2 [M + H]^+^. [^1^H]-NMR (D_2_O) δ: 1.40 (d, *J* = 6.8 Hz, 3H, CH_3_); 3.47–3.52 (m, 4H, 2CH_2_); 4.54 (q, *J* = 6.8 Hz, 1H, CH); 7.21–7.40 (m, 5H, Ar) ppm. [^13^C]-NMR (D_2_O) δ: 22.54 (CH_3_); 42.67 (2CH_2_); 52.84 (CH); 125.63 (CH_Ar_); 128.07 (CH_Ar_); 129.13 (CH_Ar_); 142.06 (C_Ar_); 159.03 (C=N) ppm.

*N*-(3-Phenylpropyl)-4,5-dihydro-1*H*-imidazol-2-amine hydroiodide **5**. White solid; m.p. 85–87 °C. [^1^H]-NMR (CDCl_3_) δ: 1.90 (p, *J* = 7.2 Hz, 2H, CH_2_); 2.70 (t, 2H, *J* = 7.9 Hz, PhCH_2_); 3.34 (apparent q, *J* = 6.4 Hz, 2H, NCH_2_); 3.60 (s, 4H, CH_2_CH_2_); 7.07 (bs, 1H, NH), 7.12–7.26 (m, 5H, Ar); 7.63 (s, 1H, NH) ppm. [^13^C]-NMR (CDCl_3_) δ: 30.68 (CH_2);_ 32.63 (CH_2_); 42.57 (CH_2_); 43.39 (CH_2_); 126.22 (CH_Ar_); 128.54 (CH_Ar_); 140.62 (C_Ar_); 159.67 (C=N) ppm. ESI-HRMS (*m/z*) [M + H]^+^: calculated for C_12_H_18_N_3_^+^ 204.1495; found 204.1498.

*N*-Benzyl-*N*-methyl-4,5-dihydro-1*H*-imidazol-2-amine hydroiodide **6**. Purification by flash chromatography (CH_2_Cl_2_/CH_3_OH/NH_3_ 80:20:1 as eluent). White solid; m.p. 150–153 °C. [^1^H]-NMR (D_2_O) δ: 2.89 (s, 3H, NCH_3_); 3.61 (s, 4H, CH_2_CH_2_); 4.42 (s, 2H, CH_2_Ph); 7.15–7.40 (m, 5H, Ar) ppm. [^13^C]-NMR (D_2_O) δ: 36.38 (CH_3_); 43.14 (2CH_2_); 54.29 (CH_2_Ph); 127.05 (CH_Ar_); 128.25 (CH_Ar_); 129.11 (CH_Ar_); 134.71 (C_Ar_); 160.548 (C=N) ppm. ESI-HRMS (*m/z*) [M + H]^+^: calculated for C_11_H_16_N_3_^+^ 190.1339; found 190.1342.

*N*-Methyl-*N*-phenethyl-4,5-dihydro-1*H*-imidazol-2-amine hydroiodide **7**. White solid; m.p. 200–204 °C. [^1^H]-NMR (D_2_O) δ: 2.80 – 2.89 (m, 5H, NCH_3_ + _Ph_CH_2_); 3.42 (s, 4H, 2CH_2_); 3.46 (t, *J* = 6.6 Hz, 2H, NCH_2_); 7.19–7.33 (m, 5H, Ar) ppm. [^13^C]-NMR (D_2_O) δ: 32.62 (CH_2_Ph); 36.19 (NCH_3_); 42.83 (N-CH_2_); 52.53 (CH_2_CH_2_); 126.98 (CH_Ar_); 128.82 (CH_Ar_); 129.05 (CH_Ar_); 138.13 (C_Ar_); 159.89 (C=N) ppm. ESI-HRMS (*m/z*) [M + H]^+^: calculated for C_12_H_18_N_3_^+^ 204.1495; found 204.1499.

1-Methyl-*N*-phenethyl-4,5-dihydro-1*H*-imidazol-2-amine hydroiodide **8**. White solid; m.p. 118–220 °C. **[**^1^H]-NMR (CDCl_3_) δ: 3.00–3.08 (m, 5H, PhCH_2_ + NCH_3_); 3.48 (s, 4H, 2CH_2_); 3.65 (apparent q, *J* = 6.8 Hz, 2H, NCH_2_); 6.96 (s, 1H, NH), 7.12–7.35 (m, 5H, Ar), 7.49 (t, *J* = 5.6, 1H, NH) ppm. [^13^C]-NMR (CDCl_3_) δ: 33.78 (CH_3_); _35_.49 (CH_2_); 41.04 (CH_2);_ 45.22 (CH_2_); 50.11 (CH_2_); 126.79 (CH_Ar_); 128.67 (CH_Ar_); 129.32 (CH_Ar_); 137.87 (C_Ar_); 158.22 (C=N) ppm. ESI-HRMS (*m/z*) [M + H]^+^: calculated for C_12_H_18_N_3_^+^ 204.1495; found 204.1494.

*N*,1-Dimethyl-*N*-phenethyl-4,5-dihydro-1*H*-imidazol-2-amine hydroiodide **9**. White solid; m.p. 109–110 °C. [^1^H]-NMR (CDCl_3_) δ: 2.82 (s, 3H, CH_3_); 2.97 (t, *J* = 6.8 Hz, 2H, PhCH_2_); 3.13 (s, 3H, CH_3_); 3.55–3.62 (m, 4H, CH_2_CH_2_); 3.77 (t, *J* = 6.9 Hz, 2H, CH_2_N); 7.32 (t, *J* = 7.5 Hz, 5H, Ar), 8.34 (bs, 1H, NH) ppm. [^13^C]-NMR (CDCl_3_) δ: 33.66 (CH_2);_ 36.97 (CH_3_); 39.70 (CH_3_); 40.33 (CH_2_); 52.89 (CH_2_); 54.71 (CH_2_); 127.23 (CH-Ar); 128.92 (CH-Ar); 136.96 (C-Ar); 162.47 (C=N) ppm. ESI-HRMS (*m/z*) [M + H]^+^: calculated for C_13_H_20_N_3_^+^ 218.1652; found 218.1649.

*N*-Benzyl-*N*,1-dimethyl-4,5-dihydro-1*H*-imidazol-2-amine hydroiodide **10**. Purification by flash chromatography (CH_2_Cl_2_/CH_3_OH/NH_3_ 90:10:1 as eluent). Oil. [^1^H]-NMR (CDCl_3_) δ: 3.01 (s, 3H, CH_3_); 3.07 (s, 3H, CH_3_); 3.70–3.87 (m, 4H, CH_2_CH_2_); 4.63 (s, 2H, CH_2_Ph); 7.22–7.35 (m, 5H, Ar), 8.10 (s, 1H, NH) ppm. [^13^C]-NMR (CDCl_3_) δ: 37.03 (CH_3_); 39.72 (CH_3_); 40.53 (CH_2_); 53.14 (CH_2_); 56.60 (CH_2_); 127.20 (CH_Ar_); 128.44 (CH_Ar_); 129.25 (CH_Ar_); 133.94 (C_Ar_); 162.63 (C=N) ppm. ESI-HRMS (*m/z*) [M + H]^+^: calculated for C_12_H_18_N_3_^+^ 204.1495; found 204.1497.

*N*-Benzyl-1-methyl-4,5-dihydro-1*H*-imidazol-2-amine hydroiodide **11**. White solid; m.p. 130–133 °C. [^1^H]-NMR (CDCl_3_) δ: 3.16 (s, 3H, CH_3_); 3.58–3.66 (m, 4H, CH_2_CH_2_); 4.67 (d, *J* = 5.6 Hz, 2H, CH_2_Ph); 6.87 (s, 1H, NH); 7.25–7.38 (m, 3H, Ar); 7.49 (d, *J* = 7.2 Hz, 2H, Ar); 8.21 (s, 1H, NH) ppm. [^13^C]-NMR (CDCl_3_) δ: 33.76 (CH_3_); 41.31 (CH_2_); 46.38 (CH_2_); 50.22 (CH_2_); 128.13 (CH_Ar_); 128.34 (CH_Ar_); 129.04 (CH_Ar_); 135.63 (C_Ar_); 158.35 (C=N) ppm. ESI-HRMS (*m/z*) [M + H]^+^: calculated for C_11_H_16_N_3_^+^ 190.1339; found 190.1338.

1-Methyl-*N*-(3-phenylpropyl)-4,5-dihydro-1*H*-imidazol-2-amine hydroiodide **12**. Purification by flash chromatography (CH_2_Cl_2_/CH_3_OH/NH_3_ 90:10:1 as eluent). Gum. [^1^H]-NMR (CDCl_3_) δ: 2.02 (p, *J* = 7.6 Hz, 2H); 2.69 (t, *J* = 8.0 Hz, 2H, CH_2_); 2.97 (s, 3H, CH_3_); 3.41–3.54 (m, 4H, CH_2_CH_2_); 3.60 (t, *J* = 8.0 Hz, 2H, CH_2_); 7.10–7.25 (m, 5H, Ar) ppm. [^13^C]-NMR (CDCl_3_) δ: 30.62 (CH_2_), 32.93 (CH_2_), 33.77 (NCH_3_), 40.97 (CH_2_), 43.83 (CH_2_), 50.11 (CH_2_), 126.01 (CH_Ar_), 128.44 (CH_Ar_), 128.58 (CH_Ar_), 141.25 (C_Ar_), 157.91 (C=N) ppm. ESI-HRMS (*m/z*) [M + H]^+^: calculated for C_13_H_20_N_3_^+^ 218.1652; found 218.1654.

*N*-(4-Chlorobenzyl)-1-methyl-4,5-dihydro-1*H*-imidazol-2-amine hydroiodide **13**. White solid, m.p. 84 °C. [^1^H]-NMR (MeOD) δ: 2.96 (s, 3H, CH_3_), 3.61–3.76 (m, 4H, CH_2_CH_2_), 4.41 (s, 2H, CH_2_), 7.31 (d, *J* = 8.4 Hz, 2H, Ar), 7.35 (d, *J* = 8.4 Hz, 2H, Ar), 7.42 (s, 2H, NH) ppm. [^13^C]-NMR (MeOD) δ: 30.68 (CH_3_), 40.95 (CH_2_), 45.41 (CH_2_), 50.09 (CH_2_), 128.92 (CH_Ar_), 130.37 (CH_Ar_), 133.46 (CCl), 135.00 (C_Ar_), 158.68 (C=N), ppm. ESI-HRMS (*m/z*) [M + H]^+^: calculated for C_11_H_15_ClN_3_^+^ 224.0949; found 224.0946.

*N*-(4-Methoxybenzyl)-1-methyl-4,5-dihydro-1*H*-imidazol-2-amine hydroiodide **14**. White solid, m.p. 167 °C. [^1^H]-NMR (CDCl_3_) δ: 3.18 (s, 3H, CH_3_), 3.64 (s, 4H, CH_2_CH_2_), 3.76 (s, 3H, OCH_3_), 4.58 (d, *J* = 5.7 Hz, 2H, CH_2_), 6.38 (s, 1H, NH), 6.85 (d, *J* = 8.6 Hz, 2H, Ar), 7.39 (d, *J* = 8.6 Hz, 2H, Ar), 8.10 (s, 1H, NH), ppm. [^13^C]-NMR (CDCl_3_) δ: 33.77 (NCH_3_), 41.29 (CH_2_), 45.86 (CH_2_), 50.14 (CH_2_), 55.36 (OCH_3_), 114.38 (CH_Ar_), 127.62 (C_Ar_), 129.69 (CH_Ar_), 158.19 (C_Ar_), 159.59 (C=N) ppm. ESI-HRMS (*m/z*) [M + H]^+^: calculated for C_12_H_18_N_3_O^+^ 220.1444; found 220.1443.

*N*-(4-Fluorobenzyl)-1-methyl-4,5-dihydro-1*H*-imidazol-2-amine hydroiodide **15**. White solid, m.p. 157 °C. [^1^H]-NMR (MeOD) δ: 2.97 (s, 3H, CH_3_), 3.61 – 3.75 (m, 4H, CH_2_CH_2_), 4.42 (s, 2H, CH_2_), 7.08 (t, *J* = 8.5 Hz, 2H, Ar), 7.42 (dd, *J* = 8.5, 5.4 Hz, 2H, Ar), ppm. [^13^C]-NMR (MeOD) δ: 30.81 (CH_3_), 40.98 (CH_2_), 45.44 (CH_2_), 50.11 (CH_2_), 115.26 (d, J_C-F_ = 22 Hz, CH_Ar_), 129.12 (d, J_C-F_ = 8 Hz, CH_Ar_), 132.25 (C_Ar_), 158.62 (C=N), 162.48 (d, J_C-F_ = 244 Hz, CF), ppm. ESI-HRMS (*m/z*) [M + H]^+^: calculated for C_11_H_15_FN_3_^+^ 208.1245; found 208.1245.

*N*-(3-Chlorobenzyl)-1-methyl-4,5-dihydro-1*H*-imidazol-2-amine hydroiodide **16**. White solid, m.p. 190 °C. [^1^H]-NMR (MeOD) δ: 2.96 (s, 3H, CH_3_), 3.58–3.71 (m, 4H), 4.41 (s, 2H, CH_2_), 7.20–7.25 (m, 1H, Ar), 7.25–7.37 (m, 3H, Ar) ppm. [^13^C]-NMR (MeOD) δ: 30.56 (CH_3_), 40.93 (CH_2_), 45.45 (CH_2_), 50.06 (CH_2_), 125.25 (CH_Ar_), 126.91 (CH_Ar_), 127.74 (CH_Ar_), 130.07 (CH_Ar_), 134.35 (C-Cl), 138.59 (C_Ar_), 154.10 (C=N) ppm. ESI-HRMS (*m/z*) [M + H]^+^: calculated for C_11_H_15_ClN_3_^+^ 224.0949; found 224.0949.

*N*-(3-Methoxybenzyl)-1-methyl-4,5-dihydro-1*H*-imidazol-2-amine hydroiodide **17**. White solid, m.p. 147 °C. [^1^H]-NMR (CDCl_3_) δ: 2.95 (s, 3H, CH_3_), 3.61 (s, 4H, CH_2_CH_2_), 3.76 (s, 3H, OCH_3_), 4.59 (s, 2H, CH_2_), 6.77 (d, *J* = 6.4 Hz, 1H, NH), 7.06–7.66 (m, 4H, Ar), 8.31 (s, 1H, NH) ppm. [^13^C]-NMR (CDCl_3_) δ: 33.70 (CH_3_), 41.32 (CH_2_), 46.20 (CH_2_), 50.22 (CH_2_), 55.68 (OCH_3_), 113.67 (CH_Ar_), 114.00 (CH_Ar_), 120.27 (CH_Ar_), 129.96 (CH_Ar_), 137.43 (C_Ar_), 158.22 (C_Ar_), 159.94 (C=N), ppm. ESI-HRMS (*m/z*) [M + H]^+^: calculated for C_12_H_18_N_3_O^+^ 220.1444; found 220.1447.

*N*-(3-Fluorobenzyl)-1-methyl-4,5-dihydro-1*H*-imidazol-2-amine hydroiodide **18**. White solid, m.p. 195 °C. [^1^H]-NMR (CDCl_3_) δ: 3.19 (s, 3H, CH_3_), 3.61–3.71 (m, 4H, CH_2_CH_2_), 4.68 (s, 2H, CH_2_), 6.95 (t, *J* = 7.7 Hz, 1H, NH), 7.09 – 7.41 (m, 4H, Ar), 8.27 (bs, 1H, NH) ppm. [^13^C]-NMR (CDCl_3_) δ: 33.86 (CH_3_), 41.19 (CH_2_), 45.42 (CH_2_), 50.20 (CH_2_), 115.05 (d, *J*_CF_= 25 Hz, CH_Ar_), 123.94 (CH_Ar_), 130.45 (d, *J* = 8 Hz, CH_Ar_), 138.48 (C_Ar_), 158.11 (C=N), 162.78 (d, *J* = 246 Hz, CF) ppm.

*N*^1^-Benzyl-*N*^2^-(4,5-dihydro-1*H*-imidazol-2-yl)ethane-1,2-diamine hydroiodide **19**. White solid, m.p. 230 °C (dec). [^1^H]-NMR (MeOD) δ: 3.37 (t, *J* = 6.2 Hz, 2H, CH_2_), 3.64 (t, *J* = 6.2 Hz, 2H, CH_2_), 3.70 (s, 4H, CH_2_CH_2_), 4.34 (s, 2H, PhCH_2_), 7.40–7.47 (m, 3H, Ar), 7.59–7.64 (m, 2H, Ar) ppm. [^13^C]-NMR (MeOD) δ: 38.87 (CH_3_), 42.82 (CH_2_CH_2_), 45.73 (CH_2_), 51.20 (PhCH_2_), 128.92 (CH_Ar_), 129.49 (CH_Ar_), 129.99 (CH_Ar_), 130.69 (C_Ar_), 159.88 (C=N) ppm. ESI-HRMS (*m/z*) [M + H]^+^: calculated for C_12_H_19_N_4_^+^ 219.1604; found 219.1606.

*N*^1^-Benzyl-*N*^2^-(1-methyl-4,5-dihydro-1*H*-imidazol-2-yl)ethane-1,2-diamine hydroiodide **20**. White solid, m.p. 235 °C (dec). [^1^H]-NMR (MeOD) δ: 2.93 (s, 3H, CH_3_), 3.33 (t, *J* = 6.0 Hz, 2H, CH_2_), 3.63–3.72 (m, 6H, 3CH_2_), 4.28 (s, 2H, PhCH_2_), 7.38–7.44 (m, 3H, Ar), 7.48 – 7.54 (m, 2H, Ar) ppm. [^13^C]-NMR (MeOD) δ: 30.59 (CH_3_), 39.07 (CH_3_), 40.94 (CH_2_), 45.61 (CH_2_), 50.04 (PhCH_2_), 51.17 (CH_2_), 128.56 (CH_Ar_), 129.37 (CH_Ar_), 129.80 (CH_Ar_), 130.81 (C_Ar_), 156.18 (C=N) ppm. ESI-HRMS (*m/z*) [M + H]^+^: calculated for C_13_H_21_N_4_^+^ 233.1761; found 233.1760.

#### 2.1.2. N1-(4,5-Dihydro-1H-imidazol-2-yl)ethane-1,2-diamine 21^27^

The compound was prepared following the procedure reported by A. Bucio-Cano et al.^27^ Yellow oil, isolated then as oxalate salt. Yield: 61%; m.p. (oxalate salt): 153–154 °C. [^1^H] NMR (D_2_O) δ: 3.10 (t, *J* = 6 Hz, 2H, CH_2_); 3.44 (t, *J* = 6 Hz, 2H, CH_2_); 3.59 (s, 4H, NHCH_2_CH_2_NH_2_) ppm. [^13^C] NMR (D_2_O) δ: 38.28 (CH_2_); 39.70 (CH_2_); 42.82 (2CH_2_); 159.80 (C); 165.0 (CO Oxalate) ppm.

#### 2.1.3. N-Phenyl-4,5-dihydro-1H-imidazol-2-amine 22^28^

1-(2-Aminoethyl)-3-phenylthiourea^29^ (110 mg, 0.56 mmol) was dissolved in THF (5 ml) and a 1 M sodium hydroxide/water solution (1 eq) was added while stirring. After 0.5 h, a solution of TsCl (1–1.1 equiv) in THF (5 ml) was slowly added and the mixture was stirred for 5 h at room T. The solvent was evaporated and diethyl ether and brine were added to the residue. The layers were separated and the aqueous layer was extracted three times with diethyl ether. The combined organic layers were dried (Na_2_SO_4_), filtered and evaporated, leaving a residue which was purified by flash chromatography (CH_2_Cl_2_: MeOH: NH_3_ 80:20:1 as eluent). Compound **22** was obtained as a white solid, m.p. 137–140 °C, in 50% yields. [^1^H]-NMR (D_2_O) δ: 3.40 (s, 4H, CH_2_CH_2_), 6.94 (d, *J* = 7.2 Hz, 2H), 7.03 (t, *J* = 7.2 Hz, 1H), 7.27 (t, *J* = 8.0 Hz, 2H) ppm. [^13^C]-NMR (D_2_O) δ: 42.37 (CH_2_), 123.34 (CH_Ar_), 123.40 (CH_Ar_), 129.52 (CH_Ar_), 146.90 (C_Ar_), 161.03 (C=N) ppm.

#### 2.1.4. 1–(2-(Methylthio)-4,5-dihydro-1H-imidazol-1-yl) ethanone 23^30^

The imidazoline **1a** was acylated on one imidazoline-nitrogen atom to obtain **23** as reported by Gomez-San Juan et al.^30^ Yellow solid, yield: 100%; m.p.= 107–110 °C. [^1^H]-NMR (D_2_O) δ: 2.07 (s, 3H, SCH_3_); 2.23 (s, 3H, CH_3_); 3.76 (t, *J* = 8.8 Hz, 2H, CH_2_); 3.92 (t, *J* = 8.8 Hz, 2H, CH_2_) ppm. [^13^C]-NMR (D_2_O) δ: 14.5 (SCH_3_); 23.5 (CH_3_); 48.0 (2CH_2_); 161.5 (C=N); 171.9 (CO) ppm.

#### 2.1.5. 2-(Benzylthio)-4,5-dihydro-1H-imidazole 24 hydrobromide^31^

A mixture of commercially-available 2-imidazolidinethione (0.1 g, 0.98 mmol) and benzyl bromide (1.1 eq) in anhydrous methanol (5 ml) was heated at 65 °C for 2 h. After cooling, the solvent was removed under vacuum and the residue was treated with Et_2_O until it became solid; filtration and drying under vacuum gave the desired compound as a white solid, m.p. 181–183 °C. ESI-LC-MS (*m/z*) 193.1 [M + H]^+^ [^1^H]-NMR (D_2_O) δ: 3.81 (s, 4H, CH_2_CH_2_); 4.35 (s, 2H, CH_2_); 7.31– 7.42 (m, 5H, Ar) ppm. [^13^C]-NMR (D_2_O) δ: 35.15 (CH_2_Ph); 45.24 (2CH_2_); 128.31 (CH_Ar_); 128.72 (CH_Ar_); 129.10 (CH_Ar_); 134.12 (C_Ar_); 169.69 (C=N) ppm.

### 2.2. CA activation

An Sx.18Mv-R Applied Photophysics (Oxford, UK) stopped-flow instrument has been used to assay the catalytic activity of various CA isozymes for CO_2_ hydration reaction^32^. Phenol red (at a concentration of 0.2 mM) was used as indicator, working at the absorbance maximum of 557 nm, with 10 mM Hepes (pH 7.5, for α-CAs)^33–37^ as buffers, 0.1 M NaClO_4_ (for maintaining constant ionic strength), following the CA-catalysed CO_2_ hydration reaction for a period of 10 s at 25 °C. The CO_2_ concentrations ranged from 1.7 to 17 mM for the determination of the kinetic parameters and inhibition constants. For each activator at least six traces of the initial 5–10% of the reaction have been used for determining the initial velocity. The uncatalysed rates were determined in the same manner and subtracted from the total observed rates. Stock solutions of activators (at 0.1 mM) were prepared in distilled-deionised water and dilutions up to 1 nM were made thereafter with the assay buffer. Enzyme and activator solutions were pre-incubated together for 15 min prior to assay, in order to allow for the formation of the enzyme–activator complexes. The activation constant (K_A_), defined similarly with the inhibition constant K_I_, can be obtained by considering the classical Michaelis–Menten equation (Equation (1), which has been fitted by non-linear least squares by using PRISM 3:

(1) $$v\;=v_{\text{max}}/\left\{1+\left(K_{M}/\left[S\right]\right)\left(1+{\left[A\right]}_{f}/K_{A}\right)\right\}$$

where [A]_f_ is the free concentration of activator.

Working at substrate concentrations considerably lower than K_M_ ([S] ≪K_M_), and considering that [A]_f_ can be represented in the form of the total concentration of the enzyme ([E]_t_) and activator ([A]_t_), the obtained competitive steady-state equation for determining the activation constant is given by Equation (2):

(2) $$v=v_{0}.K_{A}/\{K_{A}+{\left({\left[A\right]}_{t}-0.5\{\left({\left[A\right]}_{t}+{\left[E\right]}_{t}+K_{A}\right)-{\left({\left[A\right]}_{t}+{\left[E\right]}_{t}+K_{A}\right)}^{2}-4{\left[A\right]}_{t}.{\left[E\right]}_{t}\right)}^{1/2}\}\}$$

where v_0_ represents the initial velocity of the enzyme-catalysed reaction in the absence of activator^33–39^. This type of approach to measure enzyme–ligand interactions is in excellent agreement with recent results from native mass spectrometry measurements^40^.

## 3. Results and discussion

### 3.1. Chemistry

**Scheme 1.**
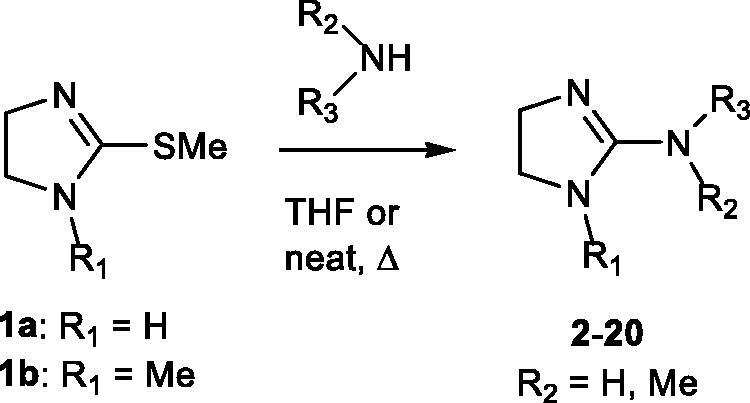
Synthesis of compounds 2-20.

2-Amino imidazolines **2**–**20** were prepared by reacting primary and secondary amines with 2-(methylthio)-4,5-dihydro-1*H*-imidazole hydroiodide **1a**^23^ or its *N*^1^-methyl derivative **1b**^24^ using tetrahydrofuran or an excess of amine as solvents (Scheme 1). The final compounds were obtained as hydroiodide salts in different yields. Synthetic details are reported in the experimental section. Reactions with 3- or 4-nitrobenzyl amine or with dibenzyl amine were unsuccessful.

**Scheme 2.**
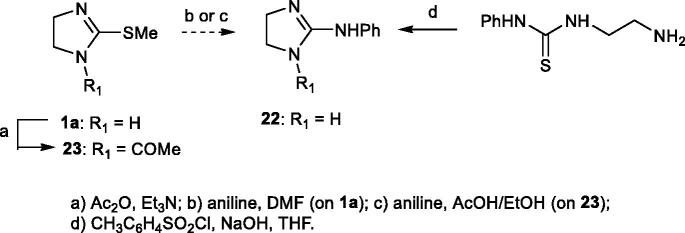
Synthesis of compounds 22 and 23.

This method failed also to give compound **22** (step b, Scheme 2), whose synthesis was therefore attempted according to the procedure of Gomez Saint-Juan (step c, Scheme 2);^30^ however, also this method was abandoned since the reaction of 1-acetyl-2-(methylthio)-4,5-dihydro-1*H*-imidazole **23** with aniline was not successful. Finally, **22** was prepared from 1–(2-aminoethyl)-3-phenylthiourea^29^ according to Heinelt et al.^28^ Compound **21**^27^ was synthesised through condensation of guanidine and ethylenediamine while **24**^31^ was prepared by reaction of imidazoline-2-thione with benzyl bromide.

### 3.2. CA activating profile

The stopped-flow method^32^ has been used for assaying the CO_2_ hydration activity catalysed by different CA isoforms; the results are expressed as K_A_ (activation constant, µM). The activating profile of CLO is reported in Table 1, in comparison with HST. CLO behaved as an activator on several CA isoforms (I, IV, VA, VII, IX, XII and XIII), with K_A_ values in the range 7.8–136 µM, while on CA II it was inactive up to a dose of 200 µM. In particular, the isoforms most sensitive to CLO were CA VII and CA XIII.

The K_A_ values of the synthesised compounds are reported in Table 3. All compounds have been tested as hydroiodide salts, with the exception of **21** (oxalate), **22** and **23** (free bases), and **24** (as HBr salt). As CLO, none of the compounds was active on hCA II at the highest tested concentration, while on the other isoforms the compounds showed K_A_ values mainly in the low-medium range, allowing to derive the following structure–activity relationships.

**Table 3.** Activation constant (K_A_) of the synthesised compounds and Clonidine (CLO) for human I, II, VA, VII and XIII Carbonic Anhydrase isoforms. 
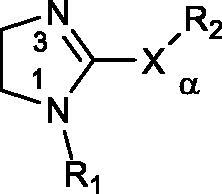

N	R_1_	X	R_2_	K_A_ (μM)^b^				
				hCA I	hCA II	hCA VA	hCA VII	hCA XIII
**1a**	H	S	–CH_3_	9.61	>150	38.3	41.9	>100
**2**	H	NH	–CH_2_Ph	4.18	>150	45.7	35.2	>100
**3**	H	NH	–(CH_2_)_2_Ph	>150	>150	16.7	18.9	>100
*S***-4**	H	NH	(*S*)–CH(Me)Ph	>150	>150	12.4	31.5	>100
*R***-4**	H	NH	(*R*)–CH(Me)Ph	>150	>150	4.92	24.2	>100
**5**	H	NH	–(CH_2_)_3_Ph	36.7	>100	9.9	11.4	24.3
**6**	H	NMe	–CH_2_Ph	>150	>150	40.5	11.0	>100
**7**	H	NMe	–(CH_2_)_2_Ph	68.6	>100	14.9	2.4	6.5
**8**	CH_3_	NH	–(CH_2_)_2_Ph	95.4	>100	14.6	16.2	31.0
**9**	CH_3_	NMe	–(CH_2_)_2_Ph	20.2	>100	14.9	2.6	36.9
**10**	CH_3_	NMe	–CH_2_Ph	30.2	>100	0.9	6.5	17.4
**11**	CH_3_	NH	–CH_2_Ph	16.9	>100	3.7	0.9	19.1
**12**	CH_3_	NH	–(CH_2_)_3_Ph	10.9	>100	17.2	3.1	10.9
**13**	CH_3_	NH	–CH_2_C_6_H_4_Cl(4)	17.7	>100	10.5	22.7	22.9
**14**	CH_3_	NH	–CH_2_C_6_H_4_OCH_3_(4)	37.6	>100	15.3	30.2	19.3
**15**	CH_3_	NH	–CH_2_C_6_H_4_F(4)	59.7	>100	9.3	17.4	24.4
**16**	CH_3_	NH	–CH_2_C_6_H_4_Cl(3)	99.2	>100	10.4	24.0	37.0
**17**	CH_3_	NH	–CH_2_C_6_H_4_OCH_3_(3)	>100	>100	13.1	29.4	14.7
**18**	CH_3_	NH	–CH_2_C_6_H_4_ F(3)	>100	>100	14.9	19.4	23.3
**19**	H	NH	–(CH_2_)_2_NHCH_2_Ph	>100	>100	11.0	41.7	20.1
**20**	CH_3_	NH	–(CH_2_)_2_NHCH_2_Ph	>100	>100	11.9	29.0	16.3
**21**	H	NH	–CH_2_CH_2_NH_2_	3.87	>150	31.2	91.6	>100
**22**	H	NH	–Ph	>150	>150	52.7	32.6	>100
**23**	COCH_3_	S	–CH_3_	12.7	>150	15.0	30.9	>100
**24**	H	S	–CH_2_Ph	>150	>150	11.1	46.7	>100
CLO	H	NH	(2,6-dichloro)Ph	76.3	>200	42.6	8.4	7.8

^a^ All compounds have been tested as HI salts, with the exception of **21** (oxalate), **22** and **23** (free bases), **24** (HBr), and CLO (HCl).

^b^ Mean from 3 different determinations (errors in the range of 5–10% of the reported values, data not shown).

#### 3.2.1. hCA I

The K_A_ value of CLO on this isoform was 76.3 µM. Removal of both chlorine atoms abolished activity, since **22** (R_2_ = Ph) was inactive when tested up to a 150 µM concentration. On the contrary, inserting a CH_2_ unit between the exocyclic N atom and the Ph ring of **22** improved the activity: in fact, **2** (K_A_ 4.18 µM), 18 times more potent than CLO, was one of the most potent compounds on this isoform among the newly synthesised analogues. The elongation of the methylene chain gave compounds less active than **2**; interestingly, a chain formed by 3 CH_2_ units was tolerated (**5**, K_A_ 36.7 µM) while a CH_2_CH_2_ chain was not (**3**, K_A_ >150 µM). Side-chain branching abolished activity (**S-4, R-4**: K_A_ >150 µM). Methylation of the exocyclic N_α_ atom was tolerated when R_2_ was a phenethyl group (**7**: K_A_ 68.6 µM) but not when R_2_ was a benzyl moiety (**6**: K_A_ >150 µM). Also the methylation of the endocyclic N_1_ atom gave contrasting results, since compounds **8** and **12** were more active than their non-methylated analogues **3** and **5**, but **11** (R_2_ = benzyl) showed a fourfold decrease of activity with respect to **2** (**11**: K_A_ 16.9; **2**: 4.18 µM). Double methylation was productive for the phenethyl analogues (compare **9** with **3**, **7** and **8**); for the benzyl analogue **10** this modification improved the activity only with respect to **6**. The aminoethyl derivative **21** (K_A_ 3.87 µM) was the most potent compound on the hCAI isoform; the addition of a benzyl moiety on the primary amine group abolished activity (**19**, K_A_ > 100 µM), and also the N^1^-methyl analogue **20** was inactive.

Aromatic substitution on the benzyl moiety did not improve the potency: in fact, while the 4-Cl derivative **13** was equiactive with **11**, a 4-OMe (**14**) or 4-F substituent (**15**) increased from 2 to 3.5 times the K_A_ values. The same substituents in the meta position reduced to a higher extent or abolished the activity. As far as the sulphur analogues **1a**, **23** and **24** are concerned, the small methyl group seemed tolerated, not the bulkier benzyl moiety (**24**, K_A_ >150 µM). The basicity of the amidine group appeared to be not crucial, since the NH and the *N*-acetyl derivatives (**1a** and **23**, respectively) were equipotent.

#### 3.2.2. hCA VA

The K_A_ value of CLO on this isoform was 42.6 µM. The removal of both chlorine atoms did not affect activity, since **22** (R_2_ = Ph, K_A_ 52.7 µM) was almost equipotent with CLO. Also the insertion of a CH_2_ unit between the exocyclic N atom and the Ph ring of **22** did not substantially modified potency (**2**, K_A_ 45.7 µM). On this isoform, the majority of the compounds showed good activating properties, with potency higher than CLO: the K_A_ values of **24**, **23**, **3**–**5**, **7**–**9**, **11** and **12** were in the range 3.7–17.2 µM.

The most potent compound was **10** (K_A_ 0.9 µM), a benzyl derivative carrying a methyl group on both N_1_ and N_α_ atoms; this compound was 47 times more active than CLO. The removal of the exocyclic N_α_-Me group decreased 4 times the activity (**11**, K_A_ 3.7 µM), while the removal of the *N*_1_-methyl group was more detrimental: as a matter of fact, compounds **6** (K_A_ 40.5 µM) and **2** (K_A_ 45.5 µM) were about 40 times less potent than **10** (K_A_ 0.9 µM). On the contrary, the degree of methylation did not substantially affect the potency of the phenylethyl and phenyl propyl derivatives, since compounds **3**, **5**, **7**, **8** and **12** had K_A_ values in the range 9.9–17.2 µM. Similarly, aromatic substitution on the benzyl moiety slightly decreased the potency without substantial modulation, the K_A_ values of compounds **13**–**18** being 2–4 times higher than **11**. Side-chain branching (compounds **S-4** and **R-4**) improved the activity on this isoform, and a small enantioselectivity was observed: the *R*-enantiomer was twice more potent as the *S*-isomer. A benzyl moiety on the terminal amino group of **21** (K_A_ 31.2 µM) increased the activity, as analogue **19** and its *N*^1^-methyl derivative **20** were about 3 times more potent than the parent compound.

As far as the sulphur derivatives are concerned, the replacement of the N_α_H moiety of **2** (K_A_ 45.5 µM) with S (**24**, K_A_ 11.1 µM) brought a fourfold improvement in activity. Acetylation of the N^1^ nitrogen was also favourable, as **23** was twice more potent than **1a**.

#### 3.2.3. hCA VII

The K_A_ value of CLO on this isoform was 8.4 µM. All the tested compounds showed activation properties on this isoform, with K_A_ values between 0.9 and 91.6 µM. The least potent compound was the primary amine **21** (K_A_ 91.6 µM), whose activity was however improved by adding a benzyl group on the terminal NH_2_ moiety (**19**, K_A_ 41.7 µM) and a methyl group on the endocyclic N atom (**20**, K_A_ 29.0 µM). The removal of chlorine atoms of CLO reduced 4 times the activity (**22**, R_2_ = Ph, K_A_ 32.6 µM) while the separation of the phenyl and N_α_H moieties by means of a CH_2_ unit did not substantially modified potency (**3**, K_A_ 35.2 µM). On the contrary, the potency increased by elongating the chain from 1 to 3 CH_2_ units (**3**, K_A_ 35.2 µM; **5**, K_A_ 11.4 µM) and by adding a methyl group on the N_α_H moiety: with the latter modification the potency of **3** (K_A_ 18.9 µM) and of **2** (K_A_ 35.2 µM) were increased 3 (**6**, K_A_ 11.0 µM) and 8 times (**7**, K_A_ 2.4 µM), respectively. Side-chain branching did not substantially affect activity, since **S-4** and **R-4** were equipotent with **2**. Methylation on the endocyclic N atom was the most effective modification in this set of molecules: as a matter of fact, with this structural change the K_A_ value of **2** (K_A_ 35.2 µM) was reduced 39 times, and **11** (K_A_ 0.9 µM) resulted in the most potent compound on this isoform. The same modification was also effective on the phenylpropyl derivative **5** (K_A_ 11.4 µM), whose activity was increased 4 times (**12**, K_A_ 3.1 µM). The double methylation on the N_1_ and N_α_ atoms gave potent compounds (**10**, K_A_ 6.5 µM and **9**, K_A_ 2.6 µM) even if the K_A_ values are, respectively, 7 and 3 times lower than that of **11**. Aromatic substitution on the benzyl moiety of **11** was detrimental for activity, as compounds **13**–**18** were 19–33 times less potent than **11**. As far as the sulphur analogues **1a**, **23** and **24** are concerned, their K_A_ values were in the range 30.9–46.7 µM, not better that the other tested 2-aminoimidazoline derivatives. Attempts to crystallise adducts of **7**, **11** and **12** with hCA VII are ongoing.

#### 3.2.4. hCA XIII

This is the isoform most sensitive to CLO among those studied (K_A_ 7.8 µM). As it happened on the hCA I isoform, the removal of both chlorine atoms, to give **22**, abolished activity. Several other compounds resulted inactive when tested at concentrations up to 100 µM, i.e. the sulphur derivatives, the polar aminoethyl derivative **21**, and all the compounds having both the N_1_ and N_α_ atoms as secondary amines, with the exception of the lipophilic phenylpropyl derivative **5** (K_A_ 24.3 µM). The activity of **21** could be restored by adding a benzyl moiety on the primary amino-group (**19**, K_A_ 20.1 µM) and a methyl group on the endocyclic N atom (**20**, K_A_ 16.3 µM). Also the methylation of the phenethyl analogue **3** on the N_α_ atom re-established activity, giving **7** (K_A_ 6.5 µM) which resulted the most potent compound of the series on this isoform. Methylation on the endocyclic N_1_ atom gave compounds **8**, **11** and **12** whose potency ranged from 10.9 to 31.0 µM, the most potent being the derivative carrying a phenylpropylamino side chain (**12**). Methylation on both N_1_ and N_α_ atoms or aromatic substitution on the benzyl moiety did not improve activity.

As far as selectivity is concerned, the two compounds showing submicromolar K_A_ values displayed also interesting selectivity profiles: **10** was more active on hCA VA with respect to hCA I (33 times), II (>100 times), VII (7 times), and XIII (19 times), while **11** showed a preference for hCA VII over hCA I (19 times), II (>100 times), VA (4 times), and XII (21 times).

## 4. Conclusions

We have synthesised a series of 2-aminoimidazolines, structurally related to Clonidine, and tested them on five different hCA isoforms (I, II, VA, VII and XIII). As the lead compound, none of the newly synthesised molecules was active on the ubiquitously expressed CA II; on the contrary, the compounds showed activity in the micromolar range on the other tested CA isoforms. Structure–activity relationships were derived, which were different on the various isoforms, suggesting that it could be possible, in this class of compounds, to find molecules, selective for a particular CA isoform. Indeed, from these preliminary modifications it has been possible to find two compounds, **10** and **11**, with a promising preference towards, respectively, CA VA and VII. Work is underway to improve both potency and selectivity, in order to find new pharmacological tool to activate specific CA isoforms in pathologies characterised by their loss of functionality.
